# Invasive *Streptococcus pyogenes* Infections in <3-Month-Old Infants in France: Clinical and Laboratory Features

**DOI:** 10.3389/fped.2020.00204

**Published:** 2020-05-06

**Authors:** Zoé Germont, Philippe Bidet, Céline Plainvert, Stéphane Bonacorsi, Claire Poyart, Valérie Biran, Alice Frérot, Albert Faye, Romain Basmaci

**Affiliations:** ^1^Service des Urgences Pédiatriques, AP-HP, Hôpital Armand Trousseau, Paris, France; ^2^Service de Microbiologie, AP-HP, Hôpital Robert-Debré, Centre National de Référence associé Escherichia coli, Paris, France; ^3^Université de Paris, IAME, INSERM, Paris, France; ^4^Service de Bactériologie, Centre National de Référence des Streptocoques, AP-HP, Groupe Hospitalier Paris Centre Cochin-Hôtel Dieu-Broca, Paris, France; ^5^INSERM, U1016, Institut Cochin, Paris, France; ^6^CNRS (UMR 8104), Paris, France; ^7^Université de Paris, Paris, France; ^8^Service de Réanimation et Pédiatrie Néonatales, Hôpital Robert-Debré, AP-HP, Paris, France; ^9^Service de Pédiatrie-Urgences, AP-HP, Hôpital Louis-Mourier, Colombes, France

**Keywords:** group A *Streptococcus*, invasive infection, children, neonatal infection, prophylaxis

## Abstract

Few data are available on invasive group A *Streptococcus* (GAS) infections (IGASIs) in infants. We described initial clinical and laboratory features and outcomes of <3-month-old infants hospitalized for an IGASI between 2007 and 2016 in France. Patients were identified from the French National Reference Centre for streptococci. IGASI was defined by the isolation of GAS from blood cultures or from other usually sterile sites. Data collection was performed by assessing the patients' hospitalization reports. Twenty-six patients (15 males; 57.7%) were included. Among 19 cases with available data, 14 (73.7%) were household contacts of a GAS infection, reaching 8/9 (88.9%) in neonates. The diagnoses were bacteremia (*n* = 18; 69.2%), pleural effusion or pneumonia (*n* = 6; 23.1%), meningitis with brain abscess (*n* = 1; 3.8%), and septic arthritis (*n* = 1; 3.8%). Fever (*n* = 10; 38.5%), hemodynamic disorders (*n* = 11; 42.3%), respiratory disorders (*n* = 7; 26.9%), thrombocytopenia (*n* = 7; 26.9%), and neutropenia (*n* = 5; 19.2%) were frequently observed. The main *emm*-genotype was *emm*-1 (*n* = 8; 30.8%). Thirteen (50.0%) infants have been admitted to the intensive care unit, and two (7.7%) died. Respiratory disorders, high C-reactive protein level, and the need for transfusion were significantly associated with severity. IGASI remains uncommon in <3-month-old children but leads to a high morbidity. Whether an antibiotic prophylaxis for contact neonates of a patient with GAS infection decreases the risk of infection remains to be determined.

## Introduction

Group A *Streptococcus* (GAS) is associated to various clinical presentations in children—from benign to severe invasive infections. In different countries, GAS was involved in 3–5% of bloodstream infections in children (1, 2). A rising incidence of invasive GAS infections (IGASIs) was observed in children in the USA from 0.16 to 0.37 per 1,000 admissions between 2009 and 2016 (2).

Neonatal infections are mainly caused by group B *Streptococcus* and *Escherichia coli*, whereas GAS is rarely involved (2). Very few data are available on IGASI in <3-month-old infants (3), while their potential severity requires early identification and appropriate management.

We aimed to describe the characteristics of IGASI in <3-month-old infants in France.

## Patients and Methods

### Inclusion Criteria and Data Collection

An IGASI was defined by the isolation of GAS from blood cultures or from other sites that would be normally sterile. In France, microbiology units of the whole country are encouraged to send the invasive GAS isolates to the French National Reference Centre for streptococci (CNR-Strep).

The records of the CNR-Strep were retrospectively queried to identify all the neonates and infants <90 days of life who were declared to have an IGASI between January 1, 2007, and December 31, 2016.

Then, each center was contacted and asked to send the hospitalization report to the first author, who collected the initial clinical and laboratory data and the clinical outcome (length of stay, complication, and transfer to intensive care unit) on a standardized anonymized paper case report form. Patients or their parents were not contacted to obtain additional or missing data. Severe IGASIs were defined as the presence of at least one of the following criteria: respiratory distress requiring mechanical ventilation, hemodynamic disorders requiring fluid resuscitation, or use of vasopressive amines, seizures, or hospitalization in the intensive care unit.

### Microbiological Analyses

Virulence genes (Table 1) as well as *emm* genotyping were detected in the CNR-Strep using polymerase chain reaction, as previously described (4, 5).

**Table 1:** Characteristics of patients with invasive Group A *Streptococcus* infection.

**Characteristics**	**Number (%) (*n* = 26)**
**Demographics**	
Age [median (range), in days]	38 (0; 88)
0–3 days	5 (19.2)
4–28 days	6 (23.1)
29–60 days	8 (30.8)
61–89 days	7 (26.9)
Gestational age [median (IQR), in weeks]	40 [39; 40]
Birth weight [median (IQR), in g]	3,510 (3,200; 3,800)
Birth weight [median (IQR), in percent]	66.5 (35.8–81.3)
**History**	
Intrapartum fever	3 (11.5)
ROM <37 weeks of gestation	1 (3.8)
Prolonged ROM (>18 h)	1 (3.8)
Abnormal fetal heart rate	2 (7.7)
Neonatal cardiac surgery	2 (10.4)
**Initial clinical characteristics**	
Time between first clinical sign and first consultation [median (range), in days]	0 (0; 14)
**Symptoms preceding admission**	
Fever	16 (61.5)
Difficult breathing	9 (34.6)
Poor feeding	7 (26.9)
Skin rash	5 (19.2)
Rhinitis	4 (15.4)
Hypotonia	4 (15.4)
Diarrhea	3 (11.5)
Vomiting	2 (7.7)
Seizure	1 (3.8)
**General physical examination at admission**	
Fever	10 (38.5)
Expiratory grunting	9 (34.6)
Pallor	8 (30.8)
**Pulmonary examination**	
Subcostal retraction	12 (46.2)
Abnormal pulmonary auscultation^*^	7 (26.9)
Oxygen support	5 (19.2)
Hemodynamic disorders^**^	11 (42.3)
Hypotonia	4 (15.4)
Cutaneous sign	11 (42.3)
**Laboratory findings**	
Anemia^‡^	5 (19.2)
White blood cell count [median (IQR), in G/L]	15.1 (13.1; 21.3)
Neutropenia^#^	5 (19.2)
Thrombocytopenia^†^	7 (26.9)
**C-reactive protein**	
Initial [median (IQR), in mg/L]	126 (44; 161)
Maximum [median (IQR), in mg/L]	181 (108; 261)
Hyponatremia	2 (7.7)
Hypernatremia	1 (3.8)
Hepatic cytolysis >1N^±^	2 (7.7)
Cholestasis (bilirubin >18 μmol/l or gamma glutamyl transferase >2N)^±^	2 (7.7)
Lactic acid >7 mmol/l	3 (11,5)
Renal insufficiency (serum creatinine >N for age)^±^	1 (3.8)
**Radiological findings**	
**Echocardiography**	
Pulmonary hypertension	4 (15.4)
Endocarditis	1 (3.8)
Pericardial effusion	1 (3.8)
**Chest X-ray**	
Pleural effusion	5 (19.2)
Alveolar opacities	4 (15.4)
**Microbiological findings**	
Blood culture positive for GAS	21/23 (91.3)
Cerebrospinal fluid culture positive for GAS	1/20 (5.0)
Pleural fluid culture positive for GAS	4/4 (100.0)
Joint fluid culture positive for GAS	1/1 (100.0)
**Streptococcal genotyping and virulence factors**	
*emm* 1	8 (30.8)
*emm* 3	2 (7.7)
*emm* 4	3 (11.5)
*emm* 6	1 (3.8)
*emm* 11	1 (3.8)
*emm* 12	3 (11.5)
*emm* 22	1 (3.8)
*emm* 28	1 (3.8)
*emm* 44	1 (3.8)
*emm* 53	1 (3.8)
*emm* 89	1 (3.8)
*emm* 104	2 (7.7)
*emm* 168.1	1 (3.8)
*spe*A	10 (38.5)
*spe*B	26 (100)
*spe*C	8 (30,8)
*sme*Z-1	5 (19.2)
*ssa*	7 (26.9)
*sic*	8 (30.8)

*Results are shown as number (%), except when specified*.

*IQR, interquartile range; ROM, rupture of membrane; GAS, group A Streptococcus; emm, M protein coding gene; spe A or C, Streptococcal pyrogenic exotoxin genes A and C; speB, cysteine protease SpeB gene; smeZ-1, allele 1 of streptococcal mitogenic exotoxin Z gene; ssa, streptococcal superantigen gene; sic, streptococcal inhibitor of complement gene*.

* *Decreased vesicular or sibilant murmur*.

** *Including tachycardia, bradycardia, hypotension, capillary refilling time >3 s, skin mottling, cold limb extremities, hepatomegaly*.

*From day 0 to day 7, hemoglobin <13g/dl; and from day 7, hemoglobin <10g/dl*.

# *From day 0 to day 7, leukocytes <6 G/L; and from day 7, leukocytes <1.5 G/L*.

† *Blood Platelets <150 G/L*.

± *Normal values provided by laboratories*.

### Statistical Analysis

We compared the severe and non-severe infection groups for each characteristic found in at least five patients. Fisher's exact test and Mann–Whitney *U*-test were used for comparisons of categorical and continuous variables, respectively. *P*-values < 0.05 were considered to be statistically significant. All statistical calculations were performed with R statistical package 3.3.2 (R Foundation for Statistical Computing, Vienna, Austria).

### Ethical Statement

The study has been approved by the Robert-Debré ethical committee (number 2016/318). As the data were obtained retrospectively with no involvement of the patients or their parents, no written informed consent was required by the ethical committee, in accordance with the local legislation. An information letter was sent to the parents. Data collection was approved by the French National Data Protection Commission (number 2002616v0).

## Results

### Demographical and Clinical Characteristics

Thirty-seven infants younger than 90 days old were initially identified from 25 cities and 12 regions, but medical records were not retrieved for 11 patients. The characteristics of the 26 included patients (15 males; 57.7%), from 18 cities and 10 regions, are shown in Table 1. None of them was preterm.

History about household contacts with GAS infection was available in only 19 cases. Out of these, 14 (73.7%) had a positive history: 7/19 (36.8%) were exposed to a maternal infection (bacteremia or gynecological infection), among whom four mothers were hospitalized for toxic shock syndrome and two died; 5/19 (26.3%) of the household contacts had pharyngitis/tonsillitis; and 2/19 (10.5%) had a cutaneous infection. Among the nine neonates, a positive history of household contacts was more common than in the 10 infants more than 28 days old [8/9 (88.9%) and 6/10 (60%), respectively; *p* = 0.3].

Among the 26 included patients, 18 (69.2%) had bacteremia, three (11.5%) had pleural effusions or pneumonia with bacteremia (one was complicated with endocarditis), three (11.5%) had pleural effusions without bacteremia, one (3.8%) had meningitis with brain abscess, and one (3.8%) had septic arthritis of the knee. Sixteen (61.5%) out of 26 patients had fever preceding admission, and 10/26 (38.5%) had fever at admission (Table 1). Of note, a portal of entry was identified in 7/26 (26.9%) cases (three impetigos, one burn, one scar, one insect bite, one cranial hematoma due to a traumatic delivery).

### Laboratory Characteristics

Laboratory findings, at presentation to the hospital, are shown in Table 1. High C-reactive protein (CRP) levels and hematological disorders (anemia, neutropenia, and thrombocytopenia) were commonly observed (Table 1).

Blood cultures were collected from 22/26 (84.6%) patients and grew GAS from 21/22 (95.5%). One out of 20 (5%) sampled cerebrospinal fluids (CSFs) grew GAS, and this patient developed a cerebral abscess. Four patients had positive culture from pleural fluid (one of them also had a positive blood culture), and one patient had a positive culture from joint fluid.

The genetic characteristics of the GAS strains are reported in Table 1. Three *emm*-types (*emm*-1, *emm*-4, and *emm*-12) represented more than the half of the observed genotypes (Table 1).

### Treatment and Outcome

Treatments are reported in Table 2. The most common antibiotics used were third-generation cephalosporin [*n* = 20/26 (76.9%); and 15/20 (75%) were switched to intravenous amoxicillin] and aminoglycosides [*n* = 19/26 (73.1%)]. Five out of the six children, who were not treated with a third-generation cephalosporin, received amoxicillin. Other drugs were more rarely prescribed and were always used in addition to another antibiotic: clindamycin (*n* = 3), vancomycin (*n* = 2), fosfomycin (*n* = 1), rifampin (*n* = 1), and imipenem (*n* = 1). Complications and outcomes of the 26 patients are presented in Table 2. Overall, 13/26 (50%) patients with IGASI were considered as severe and required admission to the intensive care unit. Four out of 26 (15.4%) patients had surgery following the onset of infection: drainage of a cerebral abscess; arthritis; sternotomy scar revision; and infection of burn site. One patient died 1 month after the diagnosis of IGASI, and one patient died 5 months later due to a non-GAS septic shock.

**Table 2:** Treatment and outcomes of patients with invasive group A *Streptococcus* infection.

**Characteristics**	**Number (%) (*n* = 26)**
**Treatment**	
Intravenous antibiotics	24 (92.3)
Duration [median (range), in days]	10 (3; 49
Switch to oral amoxicillin	7 (26.9)
Duration [median (range), in days]	7 (5; 90)
Total duration of intravenous + oral antibiotics [median (IQR), in days]	10 (7; 15)
Surgery	4 (15.4)
Transfusion	9 (34.6)
Fluid resuscitation	8 (26.9)
Catecholamine	6 (23.1)
**Outcome**	
Length of hospitalization stay [median (range) in days]	9 (1; 52)
Transfer to intensive care unit	13 (50.0)
Length of hospitalization stay [median (range) in days]	12 (4; 35)
**Complications**	
Septic shock	7 (26.9)
Seizures	3 (11.5)
Intratracheal intubation	8 (30.8)
Noninvasive ventilation	1 (3.8)
Death (within 6 months after the onset of infection)	2 (7.7)

*Results are shown as number (%), except when specified*.

*IQR, interquartile range*.

### Factors Associated With Severity

Patients with severe IGASI more commonly had abnormal respiratory symptoms, neutropenia, and a higher CRP level (*p* < 0.05) compared to the other patients (Table 3). *Streptococcus* genotypes and virulence genes were not significantly different between severe and non-severe IGASI (Table 3). Patients with non-severe infections had a shorter length of stay and a shorter total duration of antibiotics than patients with severe infections (Table 3).

**Table 3:** Comparison between patients with severe and non-severe IGASI.

**Characteristics**	**Severe patients *n* = 13 (%)**	**Non-severe patients *n* = 13 (%)**	***p***
**Demographics**			
Male/female	6 (46.2)	9 (69.2)	0.43
Age [median (IQR), in days]	43 (2; 54)	33 (15; 61)	0.5
≤ 28 days	5 (38.5)	3 (23.1)	0,67
Early onset infection (<3 days)	4 (30.8)	1 (7.7)	0.32
**First symptoms preceding admission**			
Fever	6 (46.2)	10 (76.9)	0.23
Difficult breathing	9 (69.2)	0 (0.0)	<0.001
Poor feeding	4 (30.8)	3 (23.1)	1
Skin rash	2 (15.4)	3 (23.1)	1
**Laboratory**			
Anemia	2 (15.4)	3 (23.1)	1
Neutropenia	5 (38.5)	0 (0.0)	0.04
Thrombocytopenia	6 (46.2)	1 (7.7)	0.07
CRP [median (IQR), in mg/L]	226 (137–260)	80 (43–116)	0.047
Positive blood culture	9 (69.2)	12 (92.3)	0.32
**Chest X-ray**			
Pleural effusion	5 (38.5)	0 (0.0)	0.04
**Streptococcal genotyping**			
*emm*1	5 (38.5)	3 (23.1)	0,67
*Spe*A	6 (46.2)	4 (30.8)	0.69
*sme*Z-1	4 (30.8)	1 (7.7)	0.32
*Ssa*	5 (38.5)	2 (15.4)	0.38
*Sic*	5 (38.5)	3 (23.1)	0.67
**Treatment**			
Transfusions	8 (61.5)	1 (7.7)	0.01
Total duration of intravenous + oral antibiotics [median (IQR), in days]	14.5 (12.3–21)	8 (7–10)	0.007
Length of stay [median (IQR), in days]	13 (11–26)	7.5 (6–9)	0.01

*IGASI, invasive group A Streptococcus infections; emm, M protein coding gene; speA and speC, Streptococcal pyrogenic exotoxin genes A and C; smeZ-1, the allele 1 of streptococcal mitogenic exotoxin Z gene; ssa, streptococcal superantigen gene; sic, streptococcal inhibitor of complement gene; CRP, C-reactive protein*.

## Discussion

In this study, we described a relatively large series of 26 cases in a 10-year period in France.

Considering the average number of births per year (~800,000) and the completeness of the French CNR-Strep, which has been estimated to an average rate of 28%, we could estimate the incidence of IGASI in <3-month-old children at 1.6/100,000 infants per year.

Although uncommon, our study has confirmed that IGASIs are frequently associated with clinical severity in <3-month-old infants. We found that bacteremia with no focal sign and pleuro-pneumonia were the most common clinical presentations, accounting for almost 90% of cases. Clinical characteristics were similar to those published in a previous review (3). Half of patients were considered as affected by a severe disease and exhibited more commonly respiratory disorders, neutropenia, and high CRP level and needed more transfusion and longer duration of antibiotics and length of stay compared to the patients with non-severe IGASI.

We observed that the *emm*-1,−4, and−12 represented more than half of the genotypes, similarly to the French epidemiology, described by Plainvert et al. (6) in patients younger than 15 years old with IGASI. However, no association was observed between severity and the genotypes or the presence of certain virulence genes, in contrast to previous studies in children (4). This may suggest an altered balance between host defenses in this group of age and GAS virulence.

Of note, 73.7% of the infants were household contacts of GAS infection, reaching 88.9% among neonates. This is in accordance with a study evaluating household transmission of invasive GAS in England; authors described that the risk of transmission of invasive GASI was substantially elevated in households after a single case, particularly for mothers and neonates during the neonatal period (risk ratio: 11.9; 95% confidence interval 2.0–70.3). In this population, the theoretical number needed to treat to prevent one secondary case using antibiotic prophylaxis was 50 compared to 271 for the overall population (7). Although not systematically applied in the United Kingdom and the Ireland, these countries recommend prophylaxis for postpartum mothers and neonates when the other develops IGASI (7). This is not recommended in France yet, and no children of our study received prophylaxis. Our study may suggest the need of an appropriate prophylaxis in the contact neonates of a GAS infection index case. However, given that our study did not allow describing whether otherwise healthy newborns in contact with people with GAS infection do not exhibit symptoms, further studies would be necessary to explore that hypothesis.

Our study has several limitations. Data collection was retrospective, and only 26 patients have been included despite a 10-year period in the whole country. Moreover, we could not describe the characteristics of 11 patients identified by the CNR-Strep due to the impossibility to obtain the medical records. Thus, our results may not be generalizable.

In conclusion, IGASI in <3-month-old infants remains uncommon, but 50% of patients are severely ill. The very high proportion of GAS infection in the household of these neonates suggests a need to assess whether a systematic prophylaxis and close follow-up allow avoiding these severe infections.

## Data Availability Statement

The datasets generated for this study are available on request to the corresponding author.

## Author Contributions

ZG conceptualized and designed the study, collected data, carried out the initial analyses, drafted the initial manuscript, and reviewed and revised the manuscript. PB, CPl, SB, CPo, VB, AFr, and AFa conceptualized and designed the study and critically reviewed the manuscript for important intellectual content. RB conceptualized and designed the study, coordinated and supervised data collection, drafted the initial manuscript, and reviewed and revised the manuscript. All authors approved the final manuscript as submitted and agree to be accountable for all aspects of the work.

## Conflict of Interest

The authors declare that the research was conducted in the absence of any commercial or financial relationships that could be construed as a potential conflict of interest.
